# Transitioning mpox emergency responses to more sustainable models may be a necessity

**DOI:** 10.11604/pamj.supp.2025.50.1.47574

**Published:** 2025-04-23

**Authors:** Allan Komakech

**Affiliations:** 1 School of Public Health, Clarke International University, Kampala, Uganda

**Keywords:** Mpox, emergency responses, Africa

## To the Editors of the Pan African Medical Journal

In August 2024, Africa Centres for Disease Control and Prevention (Africa CDC) declared the mpox epidemic on the African continent a public health emergency of continental security (PHECS) [1]. This was shortly followed by the World Health Organization´s (WHO) declaration of the disease as a public health emergency of international concern (PHEIC) [2]. Mpox continues to challenge African public health systems, with more countries affected. To combat the disease and transition out of the emergency mode of response, Africa CDC and WHO, in the joint continental preparedness and response plan, have outlined broad transition criteria to help countries towards the reduction of the burden of disease [3]. Some of these criteria include a sustained reduction in cases, minimal mortality, the set up of robust surveillance systems, and effective community engagement [3]. Furthermore, WHO also established a strategic medium-term strategy to provide an approach to countries based on community leadership and integration into existing health systems [4]. What remains largely absent in many countries is a clearly operationalized and context-specific exit strategy with timelines, thresholds, and funding plans that enable a smooth shift from emergency mode to an integration into the routine systems. The key questions are: What if the outbreak lasts for years? When do we consider integration into existing health systems? One equally critical but often overlooked consideration is the sustainability of prolonged emergency operations during outbreaks. For how long can we fund these outbreak responses? Is it sustainable? What happens now that donor aid funds have dwindled?

Emergency responses are, by nature, resource-intensive, expensive, and exhausting, especially for frontline health workers and overstretched national health systems [5]. In countries already battling multiple concurrent health threats, such as the Democratic Republic of Congo, maintaining mpox-specific emergency structures indefinitely can divert attention, energy, and funding from broader, long-term health system strengthening [6].

In some African countries in 2025, mpox outbreaks are no longer marked by rapidly escalating cases [7] or a lack of core capacities. Instead, they are increasingly defined by sporadic patterns, requiring routine integration into surveillance and response systems and not continuous emergency responses. It is time to ask whether the persistence of emergency responses may, in some contexts, be doing more harm than good. Otherwise, we risk creating fatigue among responders, delaying the integration of mpox into routine systems, and exhausting already limited public health budgets. Moreover, this is in light of the gradual reductions over the years in funding by the developed countries towards health initiatives in Africa [8]. During the COVID-19 pandemic, many countries only noticed the realities of integrating COVID-19 into their health systems once funding had been exhausted.

A country-based, transparent, well-communicated, and adaptable exit strategy is needed, one that transitions from emergency response to sustainable, long-term control and prevention efforts. Such a strategy should be data-driven, risk-based, and context-specific, aligning with the established indicators while considering local capacities and resource realities.

## Conclusion

As Africa moves forward in its fight against mpox, balancing emergency response with sustainability is crucial. While maintaining vigilance is essential, so is recognizing the toll of prolonged emergency operations. The time has come to evaluate, using clear data and context-specific indicators, when to responsibly shift from crisis mode to routine disease management.
